# Evolutionary perspectives on novel coronaviruses identified in pneumonia cases in China

**DOI:** 10.1093/nsr/nwaa009

**Published:** 2020-01-29

**Authors:** Xiaoman Wei, Xiang Li, Jie Cui

**Affiliations:** 1 CAS Key Laboratory of Molecular Virology & Immunology, Institut Pasteur of Shanghai, Chinese Academy of Sciences, China; 2 Wuhan Institute of Virology, Center for Biosafety Mega-Science, Chinese Academy of Sciences, China; 3 University of Chinese Academy of Sciences, China

Coronaviruses, such as severe acute respiratory syndrome coronavirus (SARS-CoV) and Middle East respiratory syndrome coronavirus (MERS-CoV), are highly transmissible pathogens, and they can be lethal in humans. Bats are believed to be the original sources of these lethal pathogens. In December 2019, a cluster of pneumonia cases was reported in Wuhan and a novel coronavirus (2019-nCoV, now officially named as SARS-CoV-2) has been linked to the causative agent. Early studies indicated that the new virus was genetically similar to a bat betacoronavirus, providing evidence for bat origin of the third human-infecting coronavirus in less than 20 years. We here highlight the potential for viral spillover from animals to humans and discuss evolutionary mechanisms leading to the outbreaks.

## CORONAVIRUSES: SARS-CoV AND MERS-CoV

Since the identification of the first coronavirus – infectious bronchitis virus (IBV) isolated from birds [1] – many coronaviruses have been discovered from such animals as bats, camels, cats, dogs, pigs, and whales [2]. They may cause respiratory, enteric, hepatic, or neurologic diseases with different levels of severity in a variety of hosts, including humans. Coronaviruses have positive-sense single-stranded RNAs, their genomic size are 26 to 32 kilobases, the largest for an RNA virus. And the viruses themselves appear crown-shaped under electron microscopy. Coronaviruses belong to the subfamily *Coronavirinae* in the family *Coronaviridae* in the order *Nidovirales*. *Coronavirinae* is further divided into four genera, *Alpha*-, *Beta*-, *Gamma*-, and *Deltacoronavirus*, based on their phylogenetic relationships and genomic structures [3].

Coronaviruses occasionally jump across host barriers, often with lethal consequences. The alpha- and betacoronaviruses only infect mammals and usually cause respiratory illness in humans and gastroenteritis in animals. Gamma- and deltacoronaviruses mainly infect birds, and no human infection has been reported. Six coronaviruses known to infect humans are 229E, NL63 (genus *Alpha-*), OC43, HKU1, SARS-CoV, and MERS-CoV (*Beta-*), whereas only SARS- and MERS-CoV have caused large worldwide outbreaks with fatality, others usually cause mild upper-respiratory tract illnesses. A novel coronavirus was identified in a pneumonia patient in Wuhan on January 9 of this year represents the seventh human-infecting coronaviruses.

Severe acute respiratory syndrome (SARS, induced by SARS-CoV) first emerged in Guangdong province, China in 2002 [4] and quickly spread around the world, with more than 8000 people infected and nearly 800 died. The MERS-CoV is a new member of *Betacoronavirus* and caused the first confirmed case of Middle East Respiratory Syndrome (MERS) in Saudi Arabia in 2012. Over 2000 MERS-related infections have been reported as of 2019 with a ∼34% fatality rate (https://www.who.int/).

## BAT ORIGIN OF PATHOGENIC CORONAVIRUSES

Bats are notorious for carrying many emerging or re-emerging viruses. Epidemiological investigations have shown that almost all SARS patients have a history of animal exposure prior to the disease. SARS-CoV and anti-SARS-CoV antibodies were first found in the masked palm civet (*Paguma larvata*) [5]. However, coronaviruses related to human SARS-CoV were found in horseshoe bats (genus *Rhinolophus*) in 2005, pointing to a bat origin of SARS-CoV [6]. This virus was named as SARS-related coronavirus (SARSr-CoV). Later, scientists reported two bat SARSr-CoVs could bind to both human and civet ACE2 receptors, suggesting that the Chinese horseshoe bats could serve as the natural reservoir of SARS-CoVs [7]. Additionally a five-year surveillance found highly diverse SARSr-CoVs in bats in one cave of Yunnan province, China, and these viral strains in this location contain all genetic building blocks needed to form a human SARS-CoV [8], further supporting a direct bat origin for human SARS-CoVs.

Viruses isolated from MERS patients were found to have close contact with dromedary camels, suggesting the animal origin [9]. Phylogenetic analysis indicated humans and camels were infected with the same source of MERS-CoV within a short time period [3]. More than 10 bat species have been found to harbor MERS-related coronaviruses (MERSr-CoVs), but structural divergence in viral proteins (such as spike proteins) exists between bat MERSr-CoVs and human/camel MERS-CoVs [10], with MERSr-CoVs at the base of phylogenetic tree, suggesting MERS-CoVs likely to originate from bats.

It is worthy of notice that in 2016 swine acute diarrhea syndrome was found in Guangdong province, China, with a mortality up to 90% for piglets. The causative pathogen was identified as a new coronavirus, called swine acute diarrhea syndrome coronavirus (SADS-CoV), shared 95% genomic identity with bat alphacoronavirus HKU2 [11], suggesting a bat spillover into pigs.

## NOVEL CORONAVIRUS RELATED TO OUTBREAK IN WUHAN

On December 31, 2019, a cluster of pneumonia cases was reported in the city of Wuhan in Hubei province, China. Laboratory tests identified a new coronavirus, excluding the possibility of a variety of pathogens including SARS-CoV, MERS-CoV, influenza virus, avian influenza virus, adenovirus, and other common respiratory pathogens. As of February 11, 2020, a total of 43,103 confirmed novel coronavirus (term hereafter as 2019-nCoV) cases have been reported globally, with 42,708 from China and 1017 deaths (https://www.who.int/).

Several genome sequences of the 2019-nCoV have been released in the http://virological.org/ and Global Initiative on Sharing All Influenza Data (GISAID) database (https://www.gisaid.org), by the Chinese Center for Disease Control and Prevention and others. Early published studies showed that the 2019-nCoV had 96.2% similarity to a bat betacoronavirus [12–17], thus classified such virus in subgenus *Sarbecovirus* of the *Betacoronavirus*. It has been proved that the new virus uses the same cell entry receptor – ACE2 as SARS-CoV, although it only had 79.5% genome sequence similarity to SARS-CoV [14,17]. By Jan 2, 2020, 66% patients were found to have visited the Huanan seafood market in Wuhan [18].

**Figure 1.**
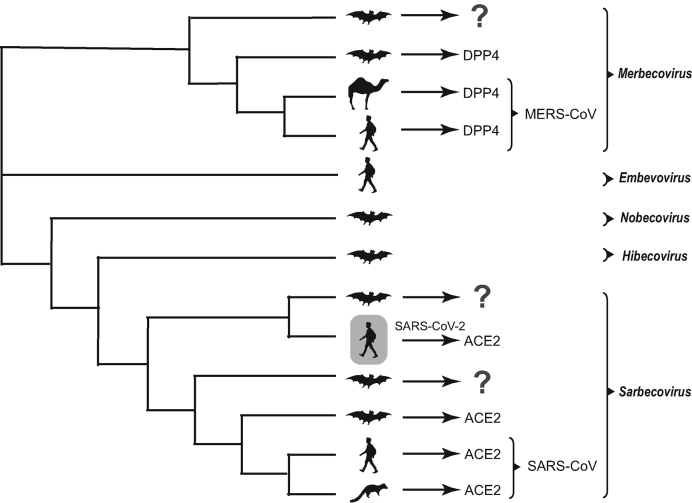
**Simplified phylogeny of 2b subgroup in *Betacoronavirus* genus**. This phylogenetic tree shows the genetic background and phylogenetics of this virus, as drawn from published findings. Subgenus was also shown for purpose of clustering only. The host figures are used to infer the origin of the viruses, and the host in gray indicates the new virus – SARS-CoV-2 discovered in Wuhan. The information of viral receptors [DPP4 (dipeptidyl peptidase-4) or ACE2 (angiotensin-converting enzyme 2)] is also shown for *Merbecovirus* and *Sarbecovirus* where ‘?’ indicates ‘an unidentified receptor’.

A simplified phylogeny is shown in Fig. 1 to illustrate the position of 2019-nCoV in the genus *Betacoronavirus*, based on the published genetic background and phylogenetics for purposes of clarity [12–17]. The 2019-nCoV has the most recent common ancestor with the neighboring bat coronaviruses, supporting the bat origin. The root of the coronavirus phylogeny was made up with other bat coronaviruses [13], which is not surprising because bats are the natural reservoir for most viruses from *Alpha-* and *Betacoronavirus* [3,19]. However, the possibility remains that unidentified intermediate hosts are responsible for direct transmission of the virus to humans. Furthermore, there is clearly a genetic gap between the novel virus and the nearest bat viruses, (Fig. 1) i.e. 3.8% genomic difference, suggesting a missing link (i.e. other hosts) may exist, given the history of SARS and MERS.

## EVOLUTIONARY MECHANISM LEADING TO NEW VIRUSES

Similar to SARS-CoV at the early stage [20], the 2019-nCoVs in Wuhan have shown mild infectivity with person-to-person transmission reported [16]. 17 nonsynonymous changes (an early sequence, ID EPI_ISL_403931, sampling collection date December 30, 2019, as reference) were observed in open reading frame 1ab (ORF1ab) (9 substitutions), spike (S) (3 substitutions), open reading frame 7a (ORF7a) (1 substitution) and open reading frame 8 (ORF8) (4 substitutions) by comparing the early released 28 viral genomes (https://bigd.big.ac.cn/ncov/#genome). Most of changes in ORF8 and S appeared in the last half of January 2020, while ORF1ab and ORF7a were from the first half. Same nonsynonymous changes were found in the S and ORF8 of a familial cluster case reported from Guangdong, i.e. L84S (Leu replaced by Ser at amino acid position 84) in three members and H49Y (His by Tyr) in two, indicating viral evolution may have occurred. However due to the limited sequence release and no functional changes given at present, adaptive evolution cannot be confirmed with these changes. Thus close monitoring of the virus's mutation, evolution, and adaptation is needed.

**Figure 2.**
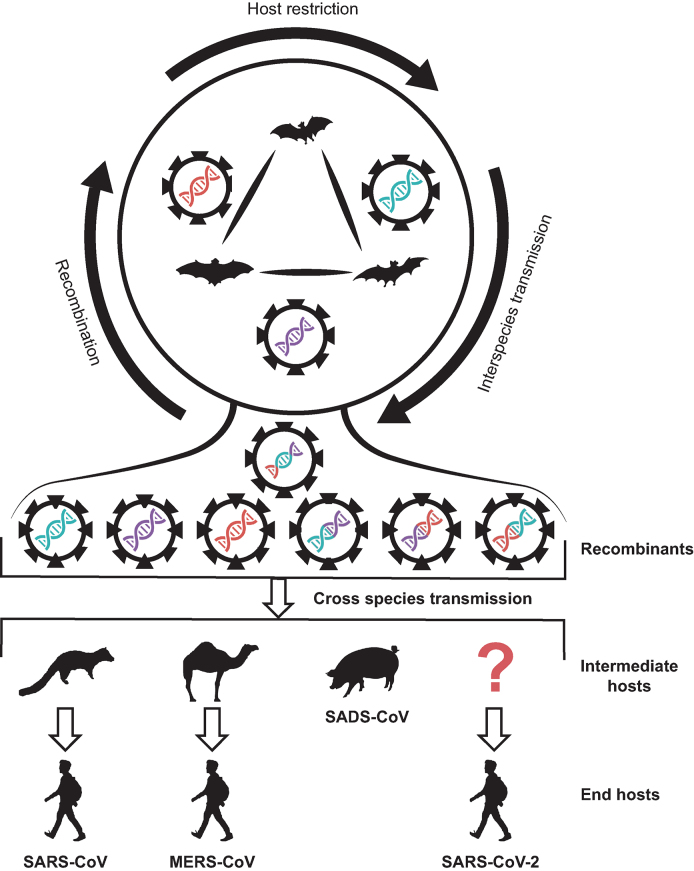
**Putative model of evolution and transmission of pathogenic coronaviruses.** Different color-coded coronaviruses on the top circle represent progenitors in natural reservoirs of different bats. Coronaviruses shown in mixed colors represent generated recombinants, which potentially lead to the cross-species transmission to other animals (intermediate hosts) or humans (end hosts).

Recombination of viruses occurs when two different parent viruses co-infect the same host cell and interact during replication, generating descendant viruses that have genes from both parental viruses. Recombination has often been associated with the expansion of the host range, increases in virulence, the evasion of host immune system and the evolution of resistance to antiviral treatments [21]. Both point mutations and recombination promote host shift for coronaviruses. For instance, there were frequent recombination events among bat SARSr-CoVs, by which progenitor of SARS-CoV could originate from [3,22].

As expected, recombination events were also involved in the emergence and evolution of MERS-CoV [23,24]. Due to the lack of genome sequences of 2019-nCoVs, recombination has not been reported so far, but merits further investigation.

Given the high prevalence of diverse SARSr-CoVs and MERSr-CoVs carried by different animals, and frequent contact histories revealed by people infected with the animal viruses, as proposed in a widely accepted hypothesis (Fig. 2) on the generation and cross-species transmission of the pathogenic coronaviruses [3], one would infer the generation of 2019-nCoVs may have similar mechanism. During the long time of co-existence of viruses and their hosts, host restriction was the dominant natural force keeping viruses circulating within the populations of the same species. However, occasionally, viruses recombine with each other in different hosts to generate new recombinants, with some of them having the potential to survive in new hosts such as humans via contacts. Recombination also occurs during circulation of different but similar viruses in human populations, also a natural process for viruses to increase genetic diversity.

## CONCLUSION AND PERSPECTIVE

Frequent human-animal contact is the major cause for viral cross-species transmission. Next-generation sequencing is a highly efficient method for rapid identification of microorganisms and for surveillance of pathogens for infectious diseases [25]. Animal models and other laboratory tests would be needed to pinpoint the causative agents. The novel coronaviruses in Wuhan likely had a bat origin, but how the human-infecting viruses evolved from bats requires further study. The human-infecting virus may become more infectious but less virulent as it continues to (co-)evolve and adapt to human hosts. Since Wuhan is one of the largest inland transportation hubs in China and the city has been closed off, it is urgently necessary to step up molecular surveillance and restrict the movement of people in and out of the affected areas promptly, in addition to closing the seafood markets. To prevent human-to-human transmission events, close monitoring of at-risk humans, including medical professionals in contact with infected patients, should also be enforced. Finally, virome projects [26] should be encouraged to help identify animal viral threats before viral spillover or becoming pandemics.
